# Major Challenges in Clinical Management of TB/HIV Coinfected Patients in Eastern Europe Compared with Western Europe and Latin America

**DOI:** 10.1371/journal.pone.0145380

**Published:** 2015-12-30

**Authors:** Anne Marie W. Efsen, Anna Schultze, Frank A. Post, Alexander Panteleev, Hansjakob Furrer, Robert F. Miller, Marcelo H. Losso, Javier Toibaro, Aliaksandr Skrahin, Jose M. Miro, Joan A. Caylà, Enrico Girardi, Mathias Bruyand, Niels Obel, Daria N. Podlekareva, Jens D. Lundgren, Amanda Mocroft, Ole Kirk, TB:HIV study group in EuroCoord

**Affiliations:** 1 Centre for Health and Infectious Disease Research (CHIP), Department of Infectious Diseases and Rheumatology, CHIP, Finsencentret, Rigshospitalet, University of Copenhagen, Copenhagen, Denmark; 2 Department of Infection and Population Health, University College London Medical School, London, United Kingdom; 3 Department of Sexual Health, Caldecot Centre, King's College Hospital, London, United Kingdom; 4 Department of HIV/TB, TB hospital 2, St. Petersburg, Russia; 5 Department of Infectious Diseases, Bern University Hospital and University of Bern, Bern, Switzerland; 6 Centre for Sexual Health and HIV Research, Mortimer Market Centre, University College London, London, United Kingdom; 7 Department of immunocompromised, Hospital J.M. Ramos Mejia, Buenos Aires, Argentina; 8 Clinical Department, Republican Research and Practical Centre for Pulmonology and TB, Minsk, Belarus; 9 Infectious Diseases Service, Hospital Clinic–IDIBAPS. University of Barcelona, Barcelona, Spain; 10 Agencia de Salud Pública de Barcelona, Barcelona, España; Programa Integrado de Investigación en Tuberculosis de SEPAR (PII-TB); Centro de Investigación Biomédica en Red de Epidemiología y Salud Pública (CIBERESP), Barcelona, Spain; 11 Department of Infectious Diseases INMI "L. Spallanzani", Ospedale L Spallanzani, Rome, Italy; 12 INSERM, ISPED, Centre Inserm U897- Epidemiologie-Biostatistique, Bordeaux, France; 13 Department of Infectious Diseases and Rheumatology, Finsencentret, Rigshospitalet, University of Copenhagen, Copenhagen, Denmark; FIOCRUZ, BRAZIL

## Abstract

**Objectives:**

Rates of TB/HIV coinfection and multi-drug resistant (MDR)-TB are increasing in Eastern Europe (EE). We aimed to study clinical characteristics, factors associated with MDR-TB and predicted activity of empiric anti-TB treatment at time of TB diagnosis among TB/HIV coinfected patients in EE, Western Europe (WE) and Latin America (LA).

**Design and Methods:**

Between January 1, 2011, and December 31, 2013, 1413 TB/HIV patients (62 clinics in 19 countries in EE, WE, Southern Europe (SE), and LA) were enrolled.

**Results:**

Significant differences were observed between EE (N = 844), WE (N = 152), SE (N = 164), and LA (N = 253) in the proportion of patients with a definite TB diagnosis (47%, 71%, 72% and 40%, p<0.0001), MDR-TB (40%, 5%, 3% and 15%, p<0.0001), and use of combination antiretroviral therapy (cART) (17%, 40%, 44% and 35%, p<0.0001). Injecting drug use (adjusted OR (aOR) = 2.03 (95% CI 1.00–4.09), prior anti-TB treatment (3.42 (1.88–6.22)), and living in EE (7.19 (3.28–15.78)) were associated with MDR-TB. Among 585 patients with drug susceptibility test (DST) results, the empiric (i.e. without knowledge of the DST results) anti-TB treatment included ≥3 active drugs in 66% of participants in EE compared with 90–96% in other regions (p<0.0001).

**Conclusions:**

In EE, TB/HIV patients were less likely to receive a definite TB diagnosis, more likely to house MDR-TB and commonly received empiric anti-TB treatment with reduced activity. Improved management of TB/HIV patients in EE requires better access to TB diagnostics including DSTs, empiric anti-TB therapy directed at both susceptible and MDR-TB, and more widespread use of cART.

## Introduction

The incidence of newly diagnosed HIV infections continues to increase in Eastern Europe (EE), whereas it has decreased in many other regions of the world [1, 2]. The situation in EE is complicated by nearly 50% of the HIV-positive patients being diagnosed at a time where HIV treatment should have been initiated (i.e. CD4<350 cells/mm^3^ or with clinical disease) and by suboptimal access to combination antiretroviral therapy (cART) [3].

Incidences of tuberculosis (TB) in EE are approximately 100 new cases per 100 000 per year in countries such as Russia, Belarus, and Latvia, and the prevalence of HIV coinfection is estimated to be 5–19% [4]. TB in HIV-positive individuals poses a diagnostic challenge in terms of lower sensitivity for smear microscopy, atypical chest radiographic appearances, and frequent extra-pulmonary localisation. TB treatment also poses a challenge due to long term high pill-burden regimens, interactions between TB medication and cART, overlapping toxicity and adherence issues, and especially in EE, high rates of multi-drug resistant TB (MDR-TB), concurrent hepatitis C coinfection, and injecting drug use (IDU) [5–7]. The prevalence of MDR-TB and extensively drug-resistant TB (XDR-TB) is particularly high, and still increasing, in EE and Central Asia [4, 8, 9]. Proportions of MDR-TB have been reported ranging from 12–35% in new TB cases to 32–77% in retreatment cases in Latvia, Russia and Belarus [6, 8, 10]. Treatment of MDR-TB and XDR-TB is both complicated and costly, and associated with high morbidity and mortality [11–14]. The extent of TB and HIV coinfection in EE is probably underestimated as many countries in this region have suboptimal diagnostic services and surveillance systems for both TB and HIV, including data on anti-TB drug resistance [8].

A previous study from our group found a three-to-five fold higher mortality among TB/HIV coinfected patients in EE compared to patients from Western Europe (WE) and Argentina. The current study builds on these previous findings and focuses on assessing potential changes over time in TB/HIV management [15]. For this analysis, we aimed to study clinical characteristics of TB/HIV patients in Europe and Latin America (LA) at TB diagnosis, identify factors associated with MDR-TB, and assess the activity of empiric anti-TB treatment regimens in relation to subsequent drug-susceptibility test (DST) results [16].

## Methods

### Study design and participants

The TB:HIV study is a prospective, observational, cohort study of TB/HIV co-infected patients aged 16 or older who were diagnosed with TB between January 1, 2011, and Dec 31, 2013, at 62 clinics in 19 different countries throughout Europe and LA (see acknowledgements). Participating sites were chosen on the basis of our previous retrospective TB:HIV collaboration on patients diagnosed in the period 2004–2006 [15]. The study protocol follow-up (FU) period is 24 months in order to analyse long-term outcome of TB disease. Here, we present analyses conducted on baseline data.

### Procedures

Data were collected on standardised case report forms (CRFs) which included demographics and information on clinical information on the TB diagnostic work up, initial anti-TB treatment, and baseline TB drug resistance. In addition, CD4 cell counts, HIV-RNA measurements, ART history, hepatitis B and C status, AIDS defining diagnoses, and malignancies were collected (www.chip.dk under TB:HIV study).

For comparative analyses, centers were divided into 4 different regions; EE (21 clinics in Belarus, Estonia, Georgia, Latvia, Lithuania, Poland, Romania, Ukraine, Russia), WE (19 clinics in Belgium, Denmark, France, Switzerland, United Kingdom), Southern Europe (SE, 9 clinics in Italy and Spain), and Latin America (LA, 13 clinics in Argentina, Chile, and Mexico). Clinics in WE, SE, and LA were included to compare EE with other European regions and another middle-income region, LA. Baseline was the date of TB diagnosis, defined as the date when TB treatment was initiated or when the first positive sample for *Mycobacterium tuberculosis (Mtb)* was obtained, whichever occurred first. TB diagnoses were classified as definite (positive culture or PCR for *Mtb*), probable (acid fast bacilli or granulomatous inflammation on sputum smear or tissue biopsy specimens), or presumptive (TB treatment initiated and not subsequently stopped because the TB diagnosis was excluded). Pulmonary TB was defined as TB localised to the lungs, larynx, or tracheobronchial tree. Extra-pulmonary TB was defined as TB localised to a single organ system (excluding lungs) and disseminated TB was defined as i) TB documented in at least two organ systems (one of which could be lungs), ii) miliary TB, or iii) isolation of *Mtb* from blood or bone marrow. MDR-TB and XDR-TB were defined according to WHO standard definitions [4].

TB treatment was categorised as RHZ-based (containing at least a rifamycin (R), isoniazid (H), and pyrazinamide (Z)) or other. Baseline resistance data were based on the first DST samples taken within one month of the baseline date.

To assess the activity of the empiric anti-TB treatment regimens, we identified the number of anti-TB drugs to which the patient’s *Mtb* isolate was subsequently found to be susceptible. All patients with DST results of cultures obtained within one month of their anti-TB treatment start date were included in this analysis; subsequent adjustments to treatment after DST results became available were not analysed. If a specific DST result was not available for a given drug, the patient was assumed to be sensitive to this drug in the primary analysis. We also assessed the hypothetical number of active drugs if all individuals were modelled as having initiated empiric therapy with RHZ plus ethambutol (E). In sensitivity analyses, we (1) calculated an alternative worst-case scenario, where missing DST results for a given drug where recoded as resistant and (2) restricted the descriptions to patients with complete resistance results (DST results available for all anti-TB drugs used in the empiric treatment regimen).

### Statistical methods

Variables of interest were stratified according to region and differences tested formally using the Kruskal-Wallis (continuous variables) or Chi-squared (proportions) tests. Logistic regression was used to identify risk factors associated with MDR-TB. Variables of interest were included in the multivariable model based on clinical plausibility and/or previous observations [14]. Details of the categorization of variables for the multivariable model are shown in the web appendix, in accordance with STROBE guidelines [17].

In order to compare results from the current analysis to findings from the retrospective HIV/TB study, data were re-analysed using the present study definitions [15]. Variables relevant for this comparison were decided upon a priori, and proportions compared using a z-test. All statistical analyses were performed using SAS (Statistical Analysis Software, Cary, NC, USA, version 9.3).

### Ethical approvals

Data were obtained from the patients’ medical records or through database exchange using HICDEP format (www.hicdep.org). The study includes clinics (N = 62) and participants across many European countries, and each participating site has a contractual obligation to ensure that data collection and sharing is done in accordance with national legislation; each site principal investigator either maintains appropriate documentation from an ethical committee (if required by law) or has a documented written statement to say that this is not required. Further, the coordinating center (CHIP) holds written copies of the participating sites ethical approvals. In specific, the study obtained ethics approval and participants gave informed consent before taking part:

Le Comité d’Ethique du C.H.U Saint-Pierre, Le Numéra registre AK/12-03-28/4128, Comité Ético Cientifico del servicio de Salud Metropolitano Centra, Chil, certificado 452/11, De Videnskabsetiske Komiteer i Region Hovedstaden Journal nr.: H-3-2011-095, United Kingdom national ethics approval Reference nr. 11/LO/0713 & R&D Reference nr. CSP 75430 for UK sites: Mortimor Market Centre, London, Imperial College Healthcare, London, St. Marys Hospital, London, North Manchester General Hospital, Manchester, Sheffield Teaching Hospitals, Sheffield, King´s College Hospital, London, North Middlesex University Hospital, London, Queen Elisabeth Hospital, London R&D ref. nr. SLHT/2011/UCSM/HIV/88, Leicester Royal Infirmary, Leicester. El Comité de Etica en Investigación en Salud, Hospital Paroissien Argentina 20/12/2911, Comité de Etica Iniciativa y Reflextion Bioetica Hospital Pineiro & Hospital Ramos Mejia Argentina 12/12/12, Comité de Bioética Reg. nr. 11743/11 Hospital Nacional Profesor Alejandro Posdas Argentina, Comité Institucional de Ètica de la Investigacion en Salud del Nino u del Adulto Cordoba 16/12/2012 Hospital Rawson, Comité de Etica La Plata 12/11/12 Hospital Sa Juan de Dios Argentina, Comité de Ética en Investigacion Buenos Aires 18/11/12 Hospital Santojani Argentina & 11/05/2012 Hospital Fracisco Javier Muniz Argentina, Docencia e Investigación 15/06/12 Jujuy Argentina, Institutional Review Board Tbilisi Ref. nr. 12–007, Registro delle Sperimentazioni del Comitato Etico Rone nr. 24/2011 Spallanzani, Comitato Etico San Gerardo, Monza 26/05/2011, Comitato Etico Aziendale A.O.U. San Martino Genoa nr. 295 08/04/2011, Comitato Etico Indipendente Locale Policlinio Consorziale Bari nr. 624 28/09/2011, Instituto Nacional de Ciencias Médicas y Nutrición Salvador Zubirán Ref. nr 437, Comité de Ética del Hospital General Regional de León 10/10/2011, Secretario del Comité de Ensenanza, Investigacion y Ética Hospital Civil de Guadalajara Invest. Nr. 038/12, Comitato Etico Spedali Civili Brescia 07/06/2011 nr.36; Direcció General de Regularcio Barcelona CY-ANT-2011-01, Hospital Universitario Ramón y Cajal Comité Ético de Investigación Clinica 12/07/2011, Comite Etico de Investigation Clinica de Euscadi Vitoria 02/02/2012, Comité Ético de Investigación Clínica Hospital General Universitario Gregorio Maranon Madrid SAS/3470/2009, Kantonale Ethik-Kommission Zürich EK-793, Ethical Committee of Rep. Res. And Practical Centre for Pulmonology Minsk 11/02/2011, Ethical Committee of Gomel State Medical University & Gomel Regional Centre for Hygiene, Ethical Committee for Botkin Hospital & City TB Hospital ref. nr.23, Ethical Committee for Novgorod Centre for AIDS Prevention and Control 11/10/2011, Ethican Committee of Samara State Medical University 10/10/2012, Komisja Bioetycznego Uniwersytetu Medycznego w Bialymstoku Poland R-I-00/85c/2012 –R-I-002/85/2011, Tallinn Medical Research Ethics Committee Appr. Nr. 2555, RSU Etikas Komitejas Lemums Riga Nr. E-9(2), Vilniaus Regioninis Biomedicininiu Tyrimu Etikos Komitetas Nr. 158200-07-363-90, Dr. Victor Babes Hospital Institutional Ethics Committee nr. 3889, Ethics Committee Crimean Republican AIDS Centre Nr. 462-13-08-12.

## Results

A total of 1413 patients were enrolled (Table 1). The majority of patients (72.5%) were male, with a median age of 36 years and a median CD4 cell count of 113 cells/mm^3^. Although the HIV diagnosis preceded the TB diagnosis by more than 3 months in 69% of participants, only 27% had ever received cART. More than half of all patients had disseminated TB, and 14% reported a prior episode of TB. We observed large regional variation in patient characteristics, with participants in EE more commonly reporting a history of IDU, recent incarceration, and more likely to be co-infected with hepatitis C and naïve to cART. Patients in both EE and LA were more likely to originate from the same country as they were being treated in and patients who inject drugs were less likely to have access to opioid substitution therapy (Table 1).

**Table 1:** Demographic and clinical characteristics of the study population.

characteristics of the study population							
		Total	Eastern Europe	Western Europe	Southern Europe	Latin America	
		N (%)	N (%)	N (%)	N (%)	N (%)	P-value
Total		1413 (100)	844 (100)	152 (100)	164 (100)	253 (100)	
Gender	Female	389 (27.5)	210 (24.9)	67 (44.1)	45 (27.4)	67 (26.5)	< .0001
Ethnicity^1^	White	971 (71.2)	773 (95.2)	39 (26.2)	112 (72.3)	47 (19.0)	< .0001
	Black African	123 (9.0)	0 (0)	94 (63.1)	27 (17.4)	2 (0.8)	
	Hispanic	189 (13.9)	0 (0)	4 (2.7)	8 (5.2)	177 (71.4)	
	Other	81 (5.9)	39 (4.8)	12 (8.1)	8 (5.2)	22 (8.9)	
Age	Years, median (IQR)	36 (31–43)	35 (31–40)	37 (32–48)	42 (33–48)	38 (30–45)	< .0001
Origin^2^	Country of origin same as centre	1156 (83.4)	828 (99.4)	20 (13.9)	81 (49.7)	227 (92.3)	< .0001
	Other European country	38 (2.7)	2 (0.2)	15 (10.4)	19 (11.7)	2 (0.8)	
	Any other country	192 (13.9)	3 (0.4)	109 (75.7)	63 (38.7)	17 (6.9)	
Weight^3^	Kg, median (IQR)	60 (53–68)	60 (53–68)	62 (53–67)	59 (54–70)	59 (50–70)	0.61
Hepatitis B^4^	HBsAg positive [current or previous]	76 (8.4)	48 (8.8)	11 (10.8)	8 (7.0)	9 (6.3)	0.57
	HBsAg tested—negative	830 (91.6)	498 (91.2)	91 (89.2)	107 (93.0)	134 (93.7)	
Hepatitis C^5^	HCV Ab positive [current or previous]	510 (54.0)	421 (74.4)	21 (19.6)	41 (34.5)	27 (17.7)	< .0001
	HCV RNA positive	67 (4.7)	19 (2.3)	18 (11.8)	24 (14.6)	6 (2.4)	
	HCV Ab tested—negative	435 (46.0)	145 (25.6)	86 (80.4)	78 (65.6)	126 (82.4)	
HIV Risk Group ^6^	MSM	137 (10.2)	12 (1.5)	16 (10.8)	29 (18.2)	80 (32.3)	< .0001
	IDU	589 (43.7)	502 (63.5)	9 (6.1)	45 (28.3)	33 (13.3)	
	Heterosexual	453 (33.7)	206 (26.0)	84 (56.8)	44 (27.7)	119 (48.0)	
	Other	167 (12.4)	71 (9.0)	39 (26.4)	41 (25.8)	16 (6.5)	
HIV disease	HIV+ >3 months before TB diagnosis^7^	973 (68.9)	635 (75.2)	82 (54.0)	99 (60.4)	157 (62.1)	< .0001
	CD4 count median (IQR) (cells/mm^3^) ^8^	113 (36–275)	107 (35–254)	149 (35–360)	129 (38–315)	96 (35–289)	0.12
	RNA median (IQR) (log^10^ copies /ml) ^9^	115E3 (2586 - 497E3)	165E3 (21500 - 538E3)	61000 (96 - 37E4)	49162 (213 - 443E3)	49600 (132 - 353E3)	< .0001
	Prior or Current AIDS	377 (26.7)	198 (23.5)	25 (16.5)	50 (30.5)	104 (41.1)	< .0001
HIV treatment	Naïve	1027 (72.7)	693 (82.1)	89 (58.6)	89 (54.3)	156 (61.7)	< .0001
	cART	361 (25.6)	140 (16.6)	60 (39.5)	72 (43.9)	89 (35.2)	< .0001
TB Risk Group	IDU	616 (43.6)	516 (61.1)	14 (9.2)	48 (29.3)	38 (15.0)	< .0001
	In prison in last 2 years	186 (13.2)	157 (18.6)	4 (2.6)	8 (4.9)	17 (6.7)	< .0001
	Alcohol misuse	306 (21.7)	202 (23.9)	12 (7.9)	19 (11.6)	73 (28.9)	< .0001
	TB cases in the family	125 (8.9)	62 (7.4)	9 (5.9)	11 (6.7)	43 (17.0)	< .0001
	Travel/Migration	100 (7.1)	2 (0.24)	64 (42.1)	29 (17.7)	5 (2.0)	< .0001
	Other risk factor	156 (11.0)	34 (4.0)	16 (10.5)	21 (12.8)	85 (33.6)	< .0001
	None indicated	335 (23.7)	183 (21.7)	49 (32.2)	60 (36.6)	43 (17.0)	< .0001
TB Type	Pulmonary	458 (32.4)	303 (35.9)	27 (17.9)	52 (31.7)	76 (30.2)	< .0001
	Extrapulmonary	194 (13.7)	59 (7.0)	37 (24.5)	38 (23.2)	60 (23.8)	
	Disseminated	758 (53.8)	481 (57.1)	87 (57.6)	74 (45.1)	116 (46.0)	
TB in the past^10^	Yes	187 (13.8)	111 (13.4)	14 (10.1)	21 (14.5)	41 (16.5)	0.36
	No	1172 (86.2)	716 (86.6)	124 (89.9)	124 (85.5)	208 (83.5)	
Current OST^11^	Yes	43 (8.5)	16 (3.7)	6 (66.7)	21 (48.8)	0 (0)	< .0001
	No	465 (91.5)	413 (96.3)	3 (33.3)	22 (51.2)	27 (100.0)	

^1^ 49 individuals had missing ethnicity.

^2^ 27 individuals had missing data on origin.

^3^ 785 individuals had missing data on weight.

^4^ 507 individuals had missing data on Hepatitis B.

^5^ 468 individuals had missing data on Hepatitis C.

^6^ 67 individuals had missing data on risk group. MSM = Men who have sex with men. IDU = Intravenous drug use.

^7^ 1220 (86.34%) individuals were known to be HIV-positive at baseline.

^8^ 204 individuals had missing baseline CD4 values.

^9^ 490 individuals had missing baseline RNA values.

^10^ 54 individuals had missing data on TB in the past.

^11^ OST = Opioid Substitution Therapy. The denominator is IDU (HIV) risk group. 81 individuals (of those who were IDU’s) had missing data on OST status.

**Fig 1:**
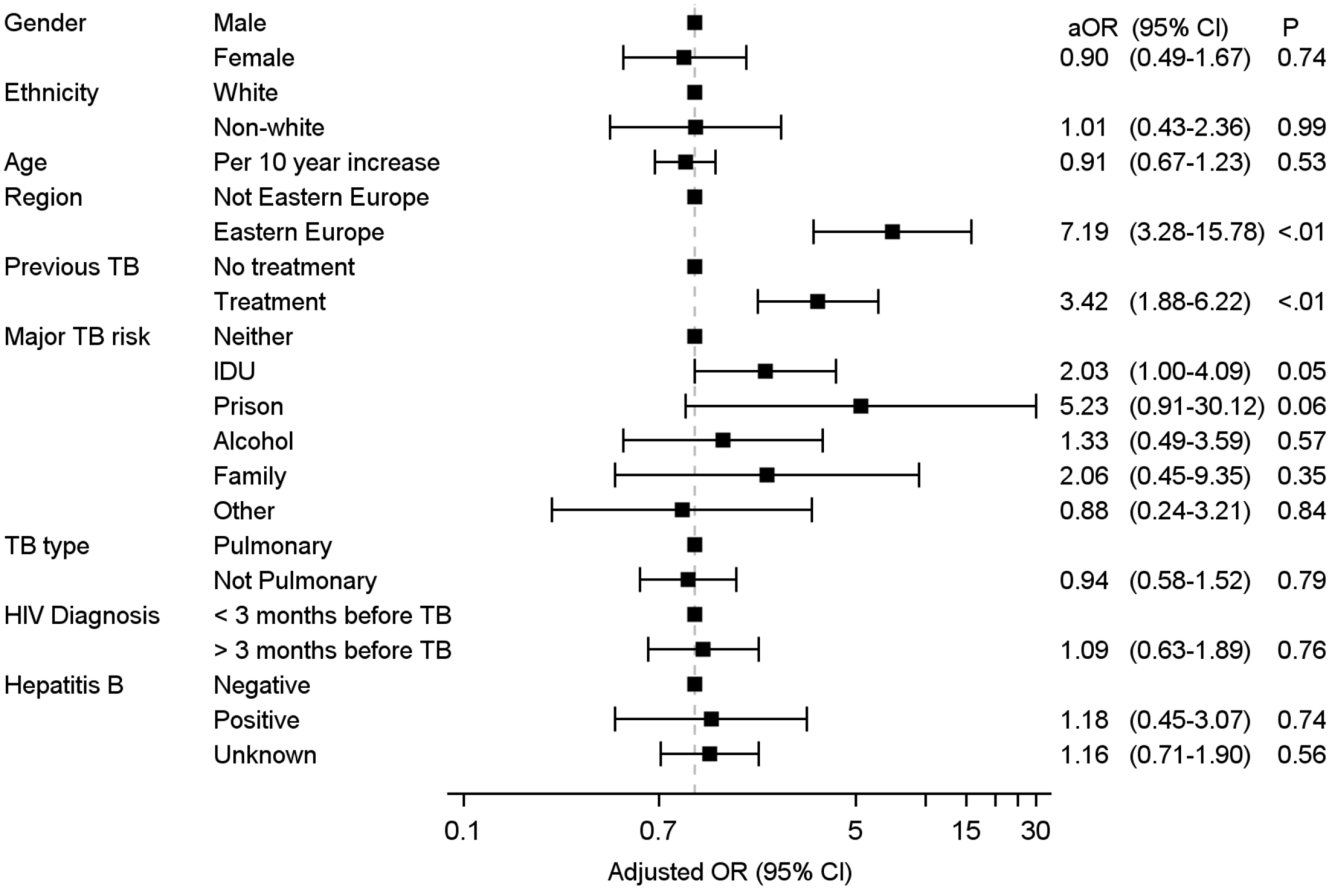
Factors associated with MDR-TB in multivariable logistic regression analysis.

The TB diagnosis was definite in approximately half of all patients, and the empiric anti-TB treatment contained RHZ in 78%. In both EE and LA, fewer patients had a definite TB diagnosis (47% and 40%, respectively, vs. 71% and 72% in WE and SE, respectively) and fewer patients in EE received empiric RHZ-containing anti-TB regimens (Table 2). Overall, 569 patients (40% of all patients, 79% of those with definite TB) had anti-TB DST performed within one month of baseline. The proportion of patients with definite TB who had DST performed was lower in EE (73% vs. 83–89%), and the prevalence of R or H resistance and MDR-TB was notably higher in EE (41%, 51% and 40%, respectively, of those tested) compared with other European regions (<10%). In LA, relatively high rates of R or H resistance and MDR-TB were encountered (18%, 25% and 15%, respectively, of those tested). The proportion of patients with no documented resistance was lower in EE (43%) compared with 89–91% in WE and SE and 74% in LA. In both EE and LA, less than half of *Mtb* isolates were tested for pyrazinamide and ethambutol resistance, and streptomycin resistance was widespread (Table 2). Of the 97 documented MDR-TB cases in EE, 27 (39.1% of 69 tested) were also resistant to kanamycin, amikacin, or capreomycin, 18 (27.7% of 65 tested) to fluoroquinolones, and 7 (14.3% of 49 tested) were XDR-TB. In multivariable models, receiving care in EE (aOR 7.2 [95% CI 3.3–15.8]), prior anti-TB treatment (aOR 3.4 [95% CI 1.9–6.2]) and history of IDU (aOR 2.0 [1.0–4.1]) were all significantly associated with MDR-TB (Fig 1).

**Table 2:** TB diagnostic status, empiric treatment regimens and drug resistance patterns.

		Total	Eastern Europe	Western Europe	Southern Europe	Latin America	
		**N (%)**	**N (%)**	**N (%)**	**N (%)**	**N (%)**	**P-value**
Diagnosis	Definite^1^	722 (51.1)	395 (46.8)	108 (71.1)	118 (72.0)	101 (39.9)	< .0001
	Probable	226 (16.0)	115 (13.6)	12 (7.9)	9 (5.5)	90 (35.6)	
	Presumptive	465 (32.9)	334 (39.6)	32 (21.1)	37 (22.6)	62 (24.5)	
Treatment^2^	RHZ based	1091 (78.2)	592 (71.3)	132 (87.4)	140 (86.4)	227 (89.7)	< .0001
	*3 drugs- RHZ only*	32 (2.9)	16 (2.7)	2 (1.5)	12 (8.6)	2 (0.9)	*<* .*0001*
	*4 drugs- RHZ + E*	868 (79.6)	416 (70.3)	118 (89.4)	120 (85.7)	214 (94.3)	*<* .*0001*
	*4 drugs- RHZ + S*	53 (4.9)	52 (8.8)	0 (0)	0 (0)	1 (0.4)	*<* .*0001*
	*4 drugs- RHZ + other*	34 (3.1)	29 (4.9)	2 (1.5)	3 (2.1)	0 (0)	*<* .*0001*
	*5 drugs- RHZ + ES*	61 (5.6)	57 (9.6)	0 (0)	2 (1.4)	2 (0.9)	*<* .*0001*
	*≥5 drugs- RHZ + other*	43 (3.9)	22 (3.7)	10 (7.6)	3 (2.1)	8 (3.5)	*<* .*0001*
	Not RHZ based	305 (21.9)	238 (28.7)	19 (12.6)	22 (13.6)	26 (10.3)	
Resistance	Tested	569 (40.3)	288 (34.1)	92 (60.5)	105 (64.0)	84 (33.2)	< .0001
	None detected^3^	363 (63.8)	123 (42.7)	82 (89.1)	96 (91.4)	62 (73.8)	< .0001
Tested for at least RH^4^	Susceptible to RH	303 (66.0)	117 (48.2)	60 (90.8)	81 (91.0)	45 (73.7)	< .0001
	R resistant/H susceptible	5 (1.1)	2 (0.8)	0 (0)	0 (0)	3 (4.9)	0.02
	H resistant/R susceptible	39 (8.5)	27 (11.1)	3 (4.6)	5 (5.6)	4 (6.6)	0.004
	Resistant (MDR-TB)	112 (24.4)	97 (39.9)	3 (4.6)	3 (3.4)	9 (14.8)	< .0001
		**N/N Tested (%)**	**N/N Tested (%)**	**N/N Tested (%)**	**N/N Tested (%)**	**N/N Tested (%)**	**P-value**
Resistance to specific drugs	Rifamycins (R)	126/496 (25.4)	105/259 (40.5)	5/71 (7.0)	3/93 (3.2)	13/73 (17.8)	< .0001
	Isoniazid (H)	163/506 (32.2)	132/259 (51.0)	6/81 (7.4)	8/99 (8.1)	17/67 (25.4)	< .0001
	Pyrazinamide (Z)	45/325 (13.9)	34/131 (26.0)	1/81 (1.2)	2/73 (2.7)	8/40 (20.0)	< .0001
	Ethambutol (E)	81/454 (17.8)	74/233 (31.8)	2/81 (2.5)	1/95 (1.1)	4/45 (8.9)	< .0001
	Streptomycin (S)	104/194 (53.6)	95/148 (64.2)	2/6 (33.3)	1/22 (4.6)	6/18 (33.3)	< .0001

^1^ Only 25 (3.5%) of those with a definite diagnosis were diagnosed by PCR alone.

^2^ 17 individuals lacked anti-TB treatment data and were excluded from the descriptions of empiric anti-TB treatment regimens. 14 of these (82.35%) came from Eastern Europe. This means that the denominator is the remaining 1396 individuals.

^3^ The denominator is everyone with a resistance test at baseline.

^4^ Of those with a resistance test, 506 (88.9%), 496 (87.2%) and 459 (80.7%) were tested for H and R and MDR-TB resistance, respectively. The denominator for these categories is the 459 individuals tested for at least RH.

Overall, only 17 (1%) did not start anti-TB treatment and 1329 (95%) started treatment within 30 days of baseline. The majority of patients initiated anti-TB treatment containing at least RHZ (78%), however, this varied substantially across regions; from 71% in EE to 90% in LA; p<0.0001 (Table 2). Of these patients, the majority initiated standard treatment with RHZ plus ethambutol (E); ranging from 70% in EE to 94% in LA. Similar trends were found when restricting analyses to patients without a history of prior TB (data not shown).

**Fig 2:**
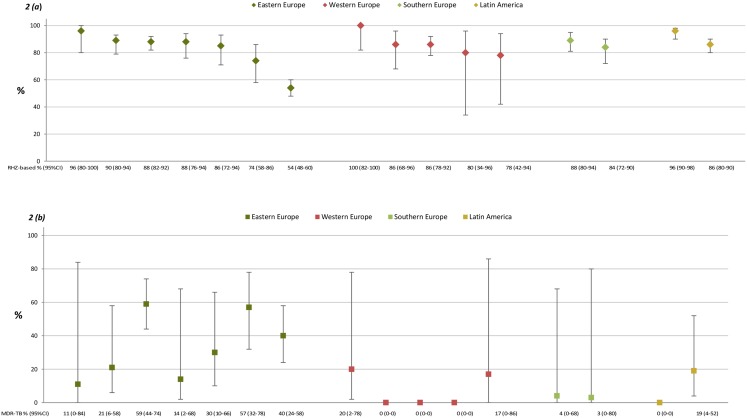
Proportion (95% CI) receiving RHZ-based empiric therapy (a) and proportion (95%CI) with MDR-TB (b) in different countries and study regions. The intraregional differences in the use of RHZ-based empiric anti-TB treatment are shown in relation to the observed MDR-TB prevalence.

In Fig 2, intraregional differences in the use of RHZ-based empiric anti-TB treatment are shown in relation to the observed MDR-TB prevalence. We observed substantial variation in the prevalence of MDR-TB in EE, ranging from 11–56% between countries with considerable uncertainty in some countries due to the small numbers of patients. The proportion of participants who initiated RHZ-based empiric anti-TB treatment appeared similar in most countries across the four regions with the exception of two countries in EE where 54% and 74% (95% CI 48–86%) of patients received such therapy. The country in which the smallest proportion of patients received RHZ-based therapy reported among the highest prevalence of MDR-TB (40%, 95% CI 24–58%). There was no evidence of a correlation between the country-wise proportions of patients initiating RHZ-based treatment and prevalences of MDR-TB (p = 0.30 and p = 0.50 for EE and WE, respectively; analysis restricted to regions with at least three data points).

**Fig 3:**
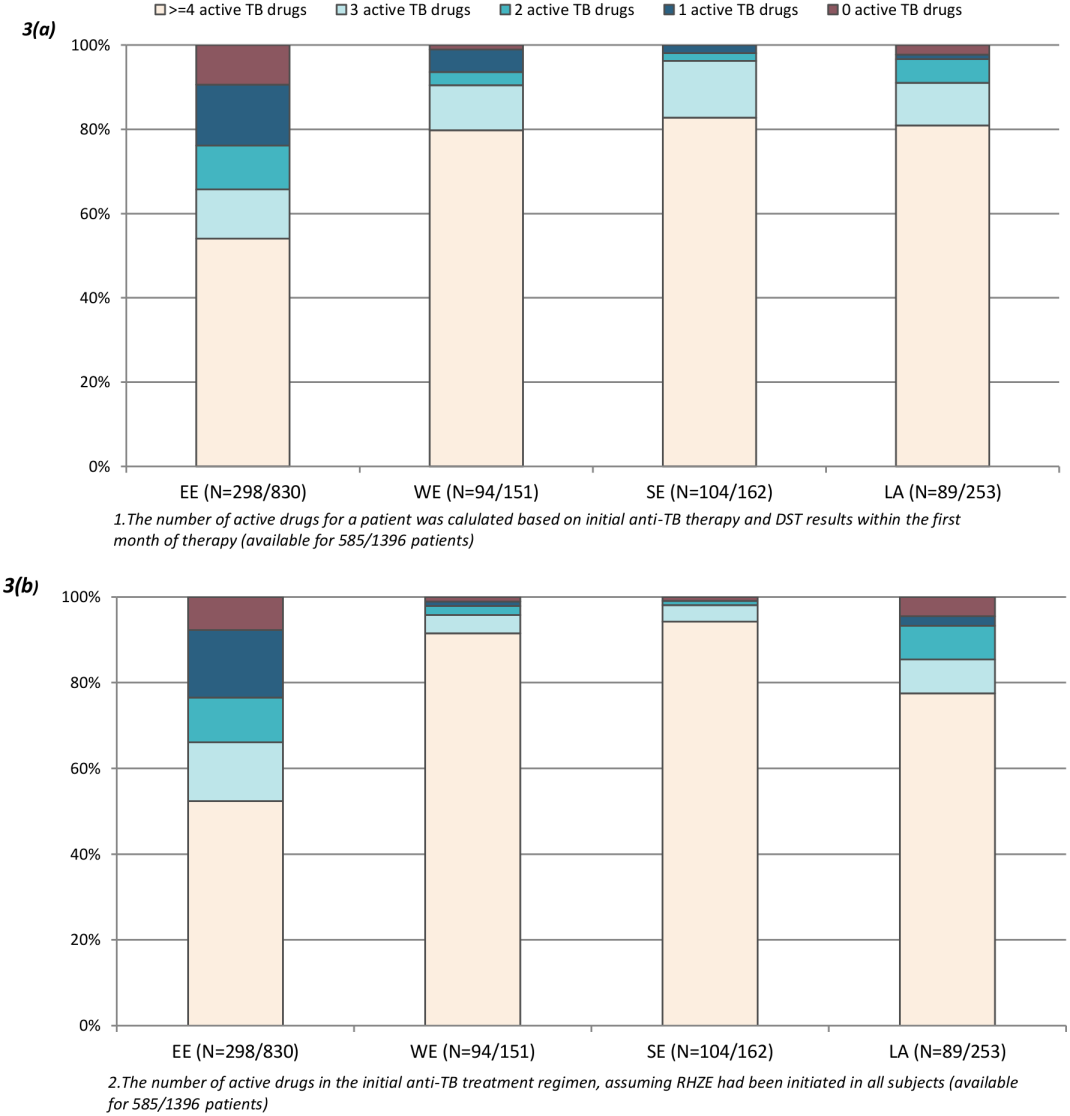
Susceptibility of empiric anti-TB treatment (a)^1^ and hypothetical susceptibility presuming RHZE had been initiated (b)^2^. ^1^The number of active drugs for a patient was calculated based on initial anti-TB therapy and DST results within the first month of therapy (available for 585/1396 patients). ^2^The number of active drugs in the initial anti-TB treatment regimen, assuming RHZE had been initiated in all subjects (available for 585/1396 patients).

For patients with DST results available, we next calculated the average number of active drugs used in the empiric anti-TB treatment regimen in relation to the subsequent DST results. The proportion of participants who received treatment with at least three active drugs ranged from 66% in EE to 90–96% in the other regions, p<0.0001, Fig 3a). Worryingly, the empiric regimen contained one or two active drugs in 25% of patients in EE, and no active drugs at all in 9%. These results did not change in sensitivity analyses restricted to individuals with full resistance profiles available. We next assumed resistance to all drugs for which no DST data were available, and the proportion receiving empiric therapy with at least three active drugs ranged from 32% and 39% in LA and EE to 75–80% in the other regions, p<0.0001, underlining that a full DST report was not available for many patients in EE and LA. Routine use of RHZE as the empiric anti-TB regimen would have had minimal impact on the proportion of patients who received at least three active drugs in all regions (Fig 3b).

We compared the data from the present study with our findings from the retrospective TB:HIV study (S1 Table). The overall number of patients on cART at baseline increased significantly from 11% to 26% (p<0.0001), the proportion of patients who initiated RHZ-containing empiric anti-TB treatment increased from 62% to 78% (p<0.0001), as did the proportion of patients diagnosed with resistance to R. The observed increases in use of RHZ-based anti-TB treatment and *Mtb* drug-resistance were largely driven by EE (data not shown). Similar results were obtained when the analysis was restricted to clinics that participated in both studies.

## Discussion

The present study documents major challenges in EE, and in our cohort more than half of the patients in EE did not have a definite TB diagnosis, approximately two thirds had no DST available and among those with a DST available, over one third had suboptimal activity of the empiric anti-TB regimen.

Levels of MDR-TB among patients from EE in our cohort are alarmingly high and appear to have increased compared to the 2004–2006 cohort, although our cohort was not designed to address time-trends in resistance prevalence [15]. In agreement with our findings, other studies have reported similarly high and increasing prevalence of MDR-TB in Belarus, Latvia and Russia [6, 10, 13, 18]. The strongest risk factor for MDR-TB in the present study was residence in EE, and the adjusted odds of having MDR-TB remained seven times higher compared to other regions. IDU and having received previous anti-TB treatment were also significantly associated with having MDR-TB, the latter being consistent with previous findings [19–21].

We found that low proportions of patients had a confirmed TB diagnosis and DST results available in both LA and EE. However, as the proportion of patients with MDR-TB was substantially lower in LA compared to EE, the majority of patients in LA were likely to have been treated for their TB disease with at least three active drugs while a large proportion of participants in EE received inadequate treatment regimens. These results emphasize the need for improved diagnostic facilities and laboratory capacity as a political and public health priority, particularly in the Eastern European region [22]. Additionally, of those patients from EE who had a DST available, only half with MDR-TB were additionally tested for XDR-TB. Rapid identification of patients infected with R resistant strains, a surrogate marker for MDR-TB, would allow for “up-front” adjustment of anti-TB treatment and thereby providing clinicians the opportunity to initiate a more accurate and effective treatment regimen. The WHO recommends that the Xpert MTB/RIF test, an automated molecular test for *Mtb* and resistance to R, should be used as the initial diagnostic test in individuals suspected of having MDR-TB or HIV-associated TB [23]. Early detection of MDR-TB is essential to reduce death rates, lower spread and prevent further development of drug resistance including development of XDR-TB, and the Xpert MTB/RIF test has been shown to be particularly effective in HIV-positive patients who more often present with smear-negative disease [24].

There are no current guidelines on specific empiric anti-TB treatment regimens in areas with high levels of anti-TB drug resistance. The World Health Organization recommends that empiric treatment includes a combination of the most potent anti-TB medication based on expert knowledge of local resistance patterns [25]. Three active drugs are generally considered as the minimum acceptable number of drugs to treat active TB disease [26, 27]. In our study, 78% of patients with a DST performed at baseline (and thus with DST results unavailable at time of initiation of the empiric treatment regimen) were initiated on anti-TB treatment with at least three drugs to which the patient’s *Mtb* isolates were subsequently not found to be resistant. In EE, around one third of the patients started less than three active drugs as part of the empiric therapy. This represents a best-case scenario and if missing resistance data for a given drug were considered as indicative of resistance to that drug, the situation would be substantially worse. This finding reflects that for many patients DST was only available for some drugs and clearly emphasizes the need for accurate and rapidly available diagnostics and DST results, as suboptimal treatment substantially increases the risk of developing further drug resistance and is associated with poorer treatment outcomes [24, 28]. Interestingly, calculating hypothetical scores (presuming RHZE treatment had been initiated in all patients) did not result in major improvements underscoring that a general recommendation of RHZE regimens is not the optimal solution in areas with high burden anti-TB drug resistance.

The challenges in EE were further documented when comparing two middle-income regions, EE and LA. The levels of anti-TB drug resistance were substantially higher in EE, as was the prevalence of inadequate empiric treatment among patients with a DST. We also observed large intraregional differences in choice of empiric therapy between countries within EE, and there was not clear correlation between usage of RHZ-based regimens and the level of MDR-TB in the individual countries. This highlights the heterogeneity in the treatment approach within EE and the present results may be used in conjunction with national-level data from countries in EE in order to inform public health policy and regional/national treatment guidelines.

Only 17% of patients in EE were on cART at time of TB diagnosis despite 75% were both known to be HIV-positive and most were severely immunosuppressed. This is in line with a recent report on very limited usage of cART among HIV-positive IDUs living in EE and calls for urgent actions to retain HIV-positive patients in EE in care and expand access to cART [29]. In our study, a large proportion of the patients were diagnosed with TB and HIV at pronounced immunodeficiency which is in agreement with results from a recent study reporting that approximately half of the patients were diagnosed late with HIV in all regions of Europe (CD4 <350/mm3 and/or AIDS events [3]. Early diagnosis of HIV infection allows for timely initiation of cART as well as preventive therapy for TB, both of which may lead to a reduction in development of active TB disease [30–32]. Taken together, these results underline the need to implement strategies to diagnose people living with HIV who remain unaware of their diagnosis.

Only 4% of IDUs in EE were receiving opioid substitution therapy although this is recommended by the WHO for IDUs infected with TB and HIV, and shown in a smaller observational study from Ukraine to increase adherence and treatment completion rates [33, 34]. Altogether, an integrated approach to patients is needed, which includes social support and access to both cART and opioid substitution therapy to ensure FU of patients and ultimately to improve outcomes [35].

Our study has several limitations. First, it is an observational study with the potential for selection bias. Secondly, clinics in the various regions were generally located in larger cities and some countries were only represented by a single clinic. As these clinics may represent centers of excellence, especially in Eastern Europe, the results presented here do not necessarily reflect the situation in all clinics in the participating countries, and the situation may well be worse in Eastern Europe. Further, DST was done at each participating clinic’s laboratory. Generally, a solid medium (Löwenstein-Jensen) was used for growing mycobacteria, but details on laboratory work were not collected and variable methodology may have biased the results. Finally, full DST results were not available for all patients. Whereas this may impact the analyses, it also reflects the clinical reality and the decision making for empiric therapy in primarily EE and LA.

In conclusion, this study documents large regional differences in clinical characteristics, levels of anti-TB drug resistance, and treatment patterns among TB/HIV coinfected patients throughout Europe and LA. Our findings demonstrate a clear need for improving and implementing more accurate and rapidly available diagnostics and for providing better empiric anti-TB treatment, particularly in EE. The focus should also be to keep patients under FU and to initiate cART when appropriate according to guidelines in order to reduce the risk of developing clinical TB disease. The long-term clinical impact of the present alarming findings will be further analysed as follow up data accumulates in this prospective study (www.cphiv.dk under TB:HIV study).

## Supporting Information

S1 Table Number of individuals receiving 0->4 active anti-TB drugs as part of their empiric treatment regimen, by region. (PDF)
