# Supplementary material for: Major Challenges in Clinical Management of TB/HIV Coinfected Patients in Eastern Europe Compared with Western Europe and Latin America
Source: PLoS One. 2015 Dec 30;10(12):e0145380. doi: 10.1371/journal.pone.0145380 (PMC4696866; doi:10.1371/journal.pone.0145380)
Supplement: S1 Table — (PDF) [file pone.0145380.s001.pdf]

S Fig. 1. Patient Flowchart

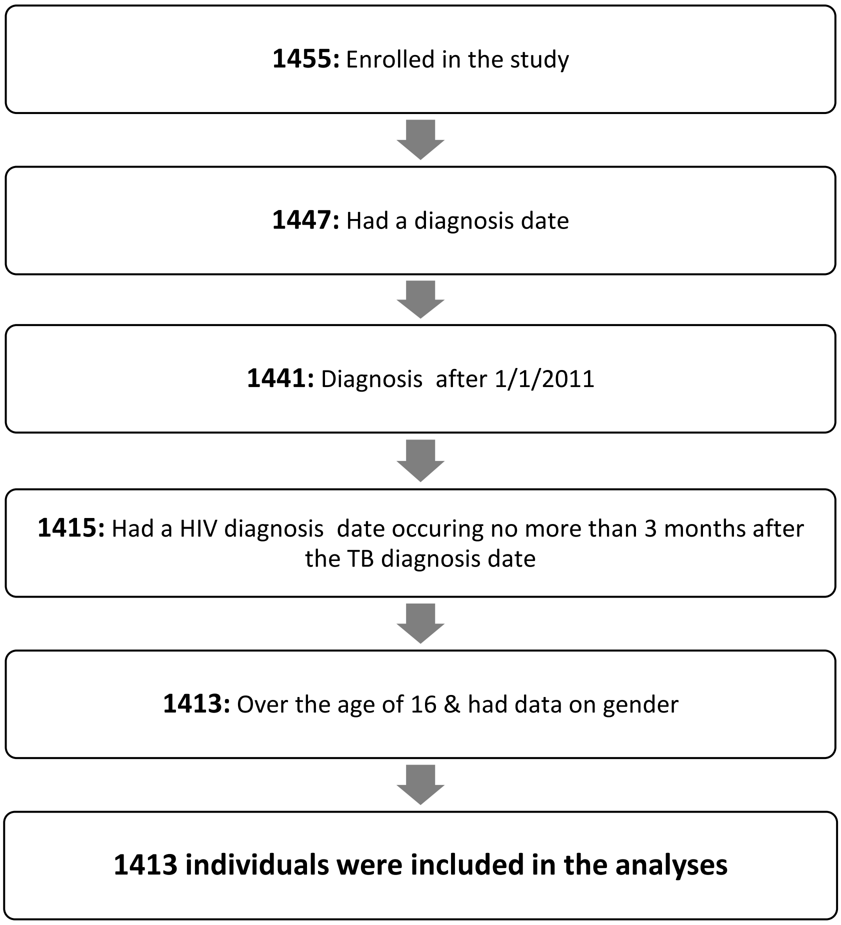

Variable Categorisation and Model building strategy (MDR-TB multivariable model)

Variable categorisation

TB risk factors were re-categorised into mutually exclusive categories to prevent multicollinearity according to the following algorithm: 1) IDU 2) Prison 3) Alcohol 4) Family 5) Other. An individual was categorised according to the 'highest' risk factor they had. Region was re-categorised into "Eastern Europe" vs "All other regions", Ethnicity into "White" vs "Non-White" and type of TB into "Pulmonary" vs "Not Pulmonary" due to small numbers of MDR-TB cases per original category (table 2). Age was modelled a continuous variable (scaled by 10). Because the proportion of missing data for the hepatitis variables was high (>30%) these were included as a separate category, in all other cases patients with missing data for any covariate were excluded from the models.

Model building strategy

Variables of interest were included in the model based on clinical knowledge and previous publications. As the aim of the analysis was to identify risk factors rather than to create a parsimonious model, all a-priori chosen variables were included in the multivariable model irrespective of their p-values. If multicollinearity was found between two variables, the variable of the least clinical interest was dropped from the multivariable model. The amount of confounding was explored by adjusting for each risk factor in turn.

S Table 1. Number of individuals receiving 0->4 active anti-TB drugs as part of their empiric treatment regimen, by region

(assuming that missing=susceptible)

	Total		Eastern Europe		Western Europe		Southern Europe		Latin America		P-value[1]
	**N**	**%**	**N**	**%**	**N**	**%**	**N**	**%**	**N**	**%**	
0 active drugs	31	*5.3*	28	*9.4*	1	*1.1*	0	*0*	2	*2.3*	<.0001
1 active drug	51	*8.7*	43	*14.4*	5	*5.3*	2	*1.9*	1	*1.1*	
2 active drugs	41	*7.0*	31	*10.4*	3	*3.2*	2	*1.9*	5	*5.6*	
3 active drugs	68	*11.6*	35	*11.7*	10	*10.6*	14	*13.5*	9	*10.1*	
>4 active drugs	394	*67.4*	161	*54.0*	75	*79.8*	86	*82.7*	72	*80.9*	
**Assuming RHZ:**	**N**	**%**	**N**	**%**	**N**	**%**	**N**	**%**	**N**	**%**	
0 active drugs	35	*6.0*	28	*9.4*	1	*1.1*	1	*1.0*	5	*5.6*	<.0001
1 active drug	78	*13.3*	66	*22.2*	3	*3.2*	1	*1.0*	8	*9.0*	
2 active drugs	62	*10.6*	47	*15.8*	4	*4.3*	4	*3.9*	7	*7.9*	
3 active drugs	410	*70.1*	157	*52.7*	86	*91.5*	98	*94.2*	69	*77.5*	
>4 active drugs	0	*0*	0	*0*	0	*0*	0	*0*	0	*0*	
**Assuming RHZE:**	**N**	**%**	**N**	**%**	**N**	**%**	**N**	**%**	**N**	**%**	
0 active drugs	29	*3.0*	23	*7.7*	1	*1.1*	1	*1.0*	4	*4.5*	<.0001
1 active drug	50	*8.6*	47	*15.8*	1	*11*	0	*0.0*	2	*2.3*	
2 active drugs	41	*7.0*	31	*10.4*	2	*2.1*	1	*1.0*	7	*7.9*	
3 active drugs	56	*9.6*	41	*13.8*	4	*4.3*	4	*3.9*	7	*7.9*	
>4 active drugs	409	*69.9*	156	*52.4*	86	*91.5*	98	*94.2*	69	*77.5*	

1. *P-values calculated using a chi-squared test*

The proportion of individuals receiving >3 active drugs was 79% overall, 66% in EE, 90% in WE, 96% in SE, and 91% in LA (p<.0001). The proportion of individuals receiving >3 active drugs who received at least RHZ was 88.5% overall, 81.1% in EE, 89.4% in WE, 94.0% in SE, and 81.5% in LA.

S Table 2. Number of individuals receiving 0-≥4 active anti-TB drugs as part of their empiric treatment regimen, by region
(assuming that missing=resistant)

	Total		Eastern Europe		Western Europe		Southern Europe		Latin America		P-value[1]
	**N**	***%***	**N**	***%***	**N**	***%***	**N**	***%***	**N**	***%***	
0 active drugs	140	*23.9*	98	*32.9*	12	*12.8*	6	*5.8*	24	*27.0*	<.0001
1 active drug	72	*12.3*	41	*13.8*	8	*8.5*	4	*3.9*	19	*21.4*	
2 active drugs	75	*12.8*	42	*14.1*	4	*4.3*	11	*10.6*	18	*20.2*	
3 active drugs	110	*18.8*	53	*17.8*	14	*14.9*	34	*32.7*	9	*10.1*	
≥4 active drugs	188	*32.1*	64	*21.5*	56	*59.8*	49	*47.1*	19	*21.4*	
**Assuming RHZ:**	**N**	***%***	**N**	***%***	**N**	***%***	**N**	***%***	**N**	***%***	
0 active drugs	170	*29.1*	118	*39.6*	13	*13.8*	8	*7.7*	31	*34.8*	<.0001
1 active drug	85	*14.5*	49	*16.4*	9	*9.6*	10	*9.6*	17	*19.1*	
2 active drugs	136	*23.3*	71	*23.8*	14	*14.9*	30	*28.9*	21	*23.6*	
3 active drugs	194	*33.2*	60	*20.1*	58	*61.7*	56	*53.9*	20	*22.5*	
≥4 active drugs	0	*0*	0	*0*	0	*0*	0	*0*	0	*0*	
**Assuming RHZE:**	**N**	***%***	**N**	***%***	**N**	***%***	**N**	***%***	**N**	***%***	
0 active drugs	146	*25.0*	99	*33.2*	13	*13.8*	7	*6.7*	27	*30.3*	<.0001
1 active drug	63	*10.8*	40	*13.4*	4	*4.3*	3	*2.9*	16	*18.0*	
2 active drugs	81	*13.9*	48	*16.1*	5	*5.3*	10	*9.6*	18	*20.2*	
3 active drugs	108	*18.5*	52	*17.5*	16	*17.0*	32	*30.8*	8	*9.0*	
≥4 active drugs	187	*32.0*	59	*19.8*	56	*59.6*	52	*50.0*	20	*22.5*	

1. *P-values calculated using a chi-squared test*

The proportion of individuals receiving ≥3 active drugs was 50.9% overall, 39.3% in EE, 74.7% in WE, 79.8% in SE, and 31.5% in LA (p<.0001).

S Table 3. Number of individuals receiving 0-≥4 active anti-TB drugs as part of their empiric treatment regimen, by region

(restricted to those individuals with complete resistance data)

	**Total**		**Eastern Europe**		**Western Europe**		**Southern Europe**		**Latin America**		**P-value[1]**
	**N**	***%***	**N**	***%***	**N**	***%***	**N**	***%***	**N**	***%***	
0 active drugs	61	*20.8*	51	*37.5*	4	*6.1*	2	*3.2*	4	*13.8*	<.0001
1 active drug	22	*7.5*	6	*4.4*	3	*4.5*	1	*1.6*	0	*0*	
2 active drugs	22	*7.5*	10	*7.4*	2	*3.0*	5	*8.1*	5	*17.2*	
3 active drugs	26	*8.9*	12	*8.8*	5	*7.6*	8	*12.9*	1	*3.4*	
≥4 active drugs	174	*59.4*	57	*41.9*	52	*78.8*	46	*74.2*	19	*65.5*	
**Assuming RHZ:**	**N**	***%***	**N**	***%***	**N**	***%***	**N**	***%***	**N**	***%***	
0 active drugs	63	*22.3*	48	*38.7*	4	*6.3*	2	*3.2*	9	*27.3*	<.0001
1 active drug	25	*8.9*	16	*12.9*	1	*1.6*	4	*6.5*	4	*12.1*	
2 active drugs	0	*0*	0	*0*	0	*0*	0	*0*	0	*0*	
3 active drugs	194	*68.8*	60	*48.4*	58	*92.1*	56	*90.3*	20	*60.6*	
≥4 active drugs	0	*0*	0	*0*	0	*0*	0	*0*	0	*0*	
**Assuming RHZE:**	**N**	***%***	**N**	***%***	**N**	***%***	**N**	***%***	**N**	***%***	
0 active drugs	61	*22.6*	48	*40.0*	4	*6.6*	2	*3.4*	7	*22.6*	<.0001
1 active drug	0	*0*	0	*0*	0	*0*	0	*0*	0	*0*	
2 active drugs	22	*8.1*	13	*10.8*	1	*1.6*	4	*6.9*	4	*12.9*	
3 active drugs	0	*0*	0	*0*	0	*0*	0	*0*	0	*0*	
≥4 active drugs	187	*69.3*	59	*49.2*	56	*91.8*	52	*89.7*	20	*64.5*	

1. *P-values calculated using a chi-squared test*

S Table 4. Comparisons between the retrospective and prospective study

	Retrospective		Prospective		
	**N**	***%***	**N**	***%***	**P-value**
**TB risk factor**[1]	1075		1413		
History of IDU	523	*48.7*	616	*43.6*	0.01
**HIV factor**[2]					
cART use at baseline	121	*11.3*	361	*25.6*	<.0001
**Diagnostics**[3]					
Confirmed	556	*51.7*	722	*51.1*	0.76
Probable	179	*16.7*	226	*16.0*	0.66
Presumptive	340	*31.6*	465	*32.9*	0.5
**Initial treatment**[4]					
RHZ-based	667	*62.1*	1091	*78.2*	<.0001
Not RHZ-based	408	*38.0*	305	*21.9*	<.0001
**Resistance**[5]					
Tested at baseline (% of total)	406	*37.8*	569	*40.3*	0.2
No documented resistance (% of tested)	304	*74.9*	363	*63.8*	<.0001
R-resistant (% of tested)	42	*10.3*	126	*22.1*	<.0001
H-resistant (% of tested)	86	*21.2*	163	*28.7*	0.009
MDR-TB (% of tested)	37	*9.1*	112	*19.7*	<.0001

1. *Discrepancy between these and previously published numbers as the denominator in the retrospective study was anyone who had indicated at least 1 risk factor, whereas the denominator in the prospective study was anyone in the study.*
2. *Discrepancy between these and previously published numbers are due to the fact that certain ARV's were automatically presumed to be given together with another ARV (and were therefore counted as 2 rather than 1), whereas this assumption was not made in the prospective study.*
3. *Discrepancy between these and previously published numbers is due to the more stringent time-frames used in the prospective study (where sample dates were required to occur within 1 month of the baseline date).*
4. *Discrepancy between these and previously published numbers is due to the fact that rifapentine is no longer included as a first-line treatment alternative.*
5. *Discrepancy between these and previously published numbers is due to the fact that intermediate resistance was classified as 'resistance' in the prospective but not in the retrospective study. More stringent time criteria were also applied.*
